# Changes in urinary metabolomic profile show the effectiveness of a nutritional intervention in children 6–12 years old: The ALINFA study

**DOI:** 10.1002/fsn3.4226

**Published:** 2024-05-15

**Authors:** Naroa Andueza, David Muñoz‐Prieto, Ana Romo‐Hualde, Marta Cuervo, Santiago Navas‐Carretero

**Affiliations:** ^1^ Department of Nutrition, Food Sciences and Physiology, Faculty of Pharmacy and Nutrition University of Navarra Pamplona Spain; ^2^ Center for Nutrition Research University of Navarra Pamplona Spain; ^3^ Navarra Institute for Health Research (IdiSNA) Pamplona Spain; ^4^ Biomedical Research Networking Center for Physiopathology of Obesity and Nutrition (CIBERObn) Institute of Health Carlos III Madrid Spain

**Keywords:** children, diet quality, metabolomics, nutrition intervention, urine samples

## Abstract

Diet plays an essential role in health and disease. Therefore, its determination is an important component of many investigations. The aim of the study was to evaluate the effect of a nutritional intervention on the urinary metabolome in children aged 6–12 years. Also, it was intended to identify biomarkers of diet quality and dietary intake. A 2‐month, randomized, controlled, parallel trial was conducted in Spanish children. The analyses focused on the ALINFA group, which followed a full‐fixed meal plan including healthy products, ready‐to‐eat meals, and healthy recipes. Diet quality was assessed by the KIDMED index and dietary intake by a food frequency questionnaire. Untargeted metabolomic analysis on urine samples was carried out, and multivariate analyses were performed for pattern recognition and characteristic metabolite identification. PLS‐DA and Volcano plot analyses were performed to identify the discriminating metabolites of this group. 12 putative metabolites were found to be the most relevant to this intervention. Most of them were products derived from protein and amino acid metabolism (N‐Ribosylhistidine, indolacrylic acid, and peptides) and lipid metabolism (3‐oxo‐2‐pentylcyclopentane‐1‐hexanoic acid methyl, Suberoyl‐L‐carnitine, and 7‐Dehydrodichapetalin E). All these metabolites decreased after the intervention, which was mainly associated with a decrease in the consumption of fatty meat and total fat, especially saturated fat. In turn, N‐Ribosylhistidine and Suberoyl‐L‐carnitine were negatively associated with diet quality, as well as able to predict the change in KIDMED index. In conclusion, the changes observed in urinary metabolome demonstrate the effectiveness of the ALINFA nutritional intervention.

## Graphical abstract

The ALINFA intervention produces changes in dietary pattern, improving the well‐being of children. This effects have been stated in phenotypical analyses (anthropometry, diet quality). Special mention to urinary concentrations of metabolites derived from protein and amino acid metabolism (N‐Ribosylhistidine, indolacrylic acid and peptides) and lipid metabolism (3‐oxo‐2‐pentylcyclopentane‐1‐hexanoic acid methyl, Suberoyl‐L‐carnitine and 7‐Dehydrodichapetalin E).

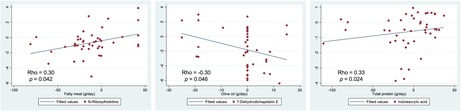

## 1 INTRODUCTION

Diet is essential for maintaining human health and preventing diseases (Grosso et al., 2020; Przybyłowicz & Danielewicz, 2022). Besides, eating habits are developed during childhood and persist into later stages of life (Brennan et al., 2021; Plaza‐Díaz et al., 2020). Therefore, the early development and consolidation of healthy eating habits is essential to preventing the onset of chronic diseases (Mikkilä et al., 2004; Shrestha & Copenhaver, 2015). For all that, it is imperative to carry out nutritional interventions aimed at this population group, children, focusing on improving the quality of their diet and the establishment of healthy eating habits.

Given the direct association between diet and health and disease, its measurement and determination are important components of a large number of investigations. At the moment, one of the most widely used methods for this are dietary questionnaires such as the food frequency questionnaire and the 24‐h recall. Despite being validated methods, they also have limitations. Since they are self‐reporting methods, this can lead to errors. Therefore, the development of biomarkers of dietary intake is essential for a more accurate assessment, adding extra information to previous methods (Clarke et al., 2022; Garcia‐Perez et al., 2017; O'Gorman et al., 2014).

In this context, metabolomics may be a key tool for identifying dietary biomarkers. Putative metabolites associated with exposure to different types of food and dietary patterns have already been identified (Grosso et al., 2020; O'Gorman et al., 2014). This discipline has also become a useful instrument in the detection of biomarkers of diseases, which could help in their prevention and early detection, allowing us to develop personalized treatments (Domínguez‐López et al., 2023; Gibbons et al., 2015). Specifically, a useful tool for exploring changes and patterns in a wide variety of metabolites is untargeted metabolomics (Andersen et al., 2014). In this sense, the urinary metabolome is the most indicated biofluid for identifying final products (Rodríguez‐Morató et al., 2018). Applying metabolomics to nutritional intervention studies allows us to study and determine the general effect of these interventions and how the components of the diet affect the metabolic pathways (Rodríguez‐Morató et al., 2018).

Some of the food biomarkers identified are related to specific dietary components or food groups such as meat (Stella et al., 2006), fruits and vegetables (Lloyd et al., 2011; May et al., 2013), seafood (Schmedes et al., 2016), milk (Brantsæter et al., 2009), and cocoa (Ibero‐Baraibar et al., 2016). Also, biomarkers of some diseases, such as type 2 diabetes mellitus, had been determined (Domínguez‐López et al., 2023; Friedrich et al., 2018).

Recently, a European multicenter study demonstrated that urinary metabolic profiles related to food components reflect adherence to the Mediterranean diet and intake of ultra‐processed foods (UPF), with both indicators of dietary quality showing an opposite pattern of association for most metabolites. Specifically, higher adherence to the Mediterranean diet was associated with higher levels of hippurate, N‐methylnicotinic acid, and urea and lower levels of sucrose, while UPF intake showed opposite associations with these four metabolites (Stratakis et al., 2022). Also, another study developed in children showed associations between BMI *z*‐score and urinary metabolic markers. Thus, a positive association was observed between the amino acid valine and this anthropometric marker. The same study also reported diet‐metabolite associations, including, most notably, urinary creatine with meat intake, urinary hippurate with vegetables, and urinary proline betaine and hippurate with fruit intake (Lau et al., 2018). Furthermore, a study conducted in Italian children showed that, compared with children with normal weight, children with obesity had higher urinary levels of glucose and 1‐methylhistidine and lower levels of xylitol, phenylacetic acid, and hydroquinone (Troisi et al., 2017).

Despite all these studies, there is still little evidence on the association between complex dietary patterns and/or pathologies and a certain metabolic urinary profile. In addition, the few studies existing to date have been carried out in very different population groups, making comparison difficult. The identification of these metabolic profiles could help to objectively evaluate the effectiveness of different nutritional interventions.

In this context, the objective of this study was to assess the effect of a 2‐month nutritional intervention aimed at improving diet quality on the urinary metabolome of children aged 6–12 years. Also, it was aimed at identifying potential key biomarkers of consumption of different nutrients and food groups and for diet quality.

## 2 METHODS

### 2.1 Study design

These new analyses are secondary to the main ALINFA study, extensively reported elsewhere (Andueza et al., 2023). The target population of this trial was 6–12‐year‐old children. Inclusion and exclusion criteria have already been described. In addition, and following the Good Clinical Practica, informed consent was signed by the tutors. The study was approved by the Research Ethics Committee of the University of Navarra (ref. 2021.027) and registered at clinicaltrials.gov (NCT05249166).

Participants were stratified by age and sex, as well as randomly allocated to the ALINFA intervention group or the control group following a 2:1 ratio.

### 2.2 Description of the nutritional intervention

Both dietary interventions, ALINFA (experimental) and control, were based on the Mediterranean dietary pattern. The main difference between the two interventions was that the experimental group received an intensive nutrition intervention, whereas the control group received only dietary advice.

Participants in the ALINFA group had to follow a full‐fixed meal plan that included healthy recipes and products, as well as ready‐to‐eat meals designed within the ALINFA consortium (made up of 3 research centers and 5 companies in the food sector). Thus, the diet was intended to provide at least 50% carbohydrates, less than 35% lipids, and 15%–20% protein per day (Andueza et al., 2023).

Participants in the control group were given nutritional advice following the recommendations of the Spanish Society of Community Nutrition (Sociedad Española de Nutrición Comunitaria (SENC), 2016).

The study was divided into a total of 4 visits: V0: information and screening; V1: start; V2: follow‐up visit; V3: end of the intervention. In visits V1 and V3, the following measurements were determined and collected: anthropometry, body composition, dietary questionnaires, and blood and urine samples. If the child was assigned to the ALINFA group, at visit V1, products as well as a record were provided. At visit V2, an interview was carried out with the participants and their tutors to evaluate the follow‐up of the intervention.

### 2.3 Measurements

Diet quality was evaluated through the KIDMED Index questionnaire (Serra‐Majem et al., 2004), which consists of 16 yes/no questions with an assigned punctuation. According to the score obtained, the quality of the diets is classified into three categories: poor quality diet (≤3 points), need to improve the dietary pattern (from 4 to 7 points), and optimal Mediterranean diet (≥8 points).

In addition, a 138‐item semiquantitative food frequency questionnaire (FFQ) validated in children was used to collect additional dietary information (Da Rocha et al., 2021), with portion sizes defined for each item. Nutrient content was determined based on the Spanish food composition database (Moreiras Tuni et al., 2015).

Anthropometric measurements, as well as body composition, were obtained under fasting conditions. The height measure was obtained with a stadiometer (Seca 220, Vogel & Halke, Germany). Body mass index (BMI) *z*‐score was assessed using the World Health Organization (WHO) classification (de Onis & Lobstein, 2010), while weight and body composition were assessed by bioimpedance (SC‐330, Tanita, Tokyo, Japan). Waist circumference was measured with an inelastic tape, and blood pressure was determined with an automatic sphygmomanometer (IntelliSense. M6, OMRON Healthcare, Hoofddorp, the Netherlands).

Blood samples were also collected under fasting conditions and subsequently processed in a standard centrifuge to obtain serum and plasma (Eppendorf 5804R, Hamburg, Germany). Glucose and lipid metabolism markers were measured in a Pentra C200 autoanalyzer (Horiba ABX Diagnostics, Montpellier, France). Inflammation marker concentrations were quantified by ELISA (DRG Instruments GmbH, Germany) in the automated ELISA processing system DSX® Dynex Technologies (Virginia, USA). Low‐density lipoprotein (LDL‐c) level concentration was calculated by the Friedewald formula (Friedewald et al., 1972).

### 2.4 Urine collection

Urine spot samples were picked up the morning of the start and end of the intervention. Samples were collected by all the volunteers in a urine container, chilled at 4°C, and then stored in vials of 650 μL at −80°C until analysis.

### 2.5 Sample preparation and high‐performance liquid chromatography‐time‐of‐flight‐mass spectrometry (HPLC‐TOF‐MS) analysis

After centrifugation of the urine samples, the supernatant was diluted with Milli Q water and transferred to a vial for the subsequent analyses through High‐Performance Liquid Chromatography (Agilent Technologies 1200, CA, USA) coupled with Mass Spectrometry (Agilent Technologies 6220 Accurate‐Mass TOF LCMS, CA, USA). Mass spectrometry operated in negative electrospray (ESI−) or positive ionization (ESI+) mode. The mobile phase consisted of water with 0.1% formic acid and acetonitrile with 0.1% formic acid. The column employed was a Zorbax SB‐C18 (15 cm × 0.46 cm; 5 μm) with a SB‐C18 precolumn (Agilent Technologies, Barcelona, Spain), following the methods previously described (Romo‐Hualde et al., 2018).

To assess the quality of this metabolomic analysis, a method already described in the literature was used (Gika et al., 2007; Llorach et al., 2009), with minor modifications (Romo‐Hualde et al., 2018).

Methanol and formic acid were purchased from Scharlau (Sentmenat, Spain), while the remaining standards were supplied by Sigma Aldrich (Steinheim, Germany).

### 2.6 Data processing and metabolite identification

XCMS Online software (https://xcmsonline.scripps.edu) was used to analyze LC–MS data by identifying and aligning features (Tautenhahn et al., 2012). First, a pairwise analysis of the control group and the ALINFA group was performed separately, in which the differences within each group at baseline and post‐intervention levels were compared. The alignment for pairwise analyses applied a 0.2‐min retention time and a 0.005 Da mass tolerance window. Next, a second‐order analysis of untargeted metabolomic data was performed by metaXCMS software for effective data reduction (Tautenhahn et al., 2011). metaXCMS was used to import the results of pairwise analyses that were subsequently filtered, realigned, and ultimately compared to identify the features of each intervention group, and the features common to both groups were determined (Venn diagram).

The metabolomic analysis was focused on the results of specific features that were only found in the ALINFA group and therefore could be considered as the discriminating metabolites of this group. Common features of two groups and specific features of the control group were disregarded. Since the control group did not receive an intensive nutritional intervention such as the ALINFA intervention, it was assumed that there would be no differences after the intervention in this group.

Metabolic identification was carried out using the Metabolomics Workbench (https://www.metabolomicsworkbench.org/), basing it on the exact mass, comparing it to the one recorded in the software (mass accuracy under 5 mDa), and also based on the provided score by the software or other databases (KEGG database – http://www.genome.jp/kegg/; HMDB database – http://www.hmdb.ca/; or Lipidmaps – http://www.lipidmaps.org/).

### 2.7 Statistical analysis

The sample size was calculated based on the primary outcome (KIDMED index), taking into account a power of 90%, and a bilateral alpha risk of 0.05, as well as a ratio of 2:1 (intervention:control). It was estimated that 40 children were needed. Since a dropout rate of 30% was established, the total sample size was estimated to be 52 children (34 in the intervention group and 18 in the control group).

Metabolic profile examination was done using MetaboAnalyst 5.0 software (http://www.metaboanalyst.ca/) (Pang et al., 2021). This analysis was performed on the characteristic metabolites of the ALINFA group, since Meta XCMS had previously identified the metabolites of this group. Prior to performing various data analysis procedures, a logarithmic transformation and monitorization by autosclaing were used to control the peak intensity. In addition, a Volcano plot analysis and PLS‐DA (Partial Least Squares – Discriminant Analysis) were performed. The evaluation, fitness, and prediction power of the PLS‐DA model were tested by a 5‐fold cross‐validation and permutation test. The most relevant metabolites were identified using the Volcano plot model. The Fold Change (FC) selected was 1.45. Metabolites with a Variable Importance in Projection (VIP) greater than 1 were selected. The *p*‐value for each metabolite was obtained from the results of the Volcano plot. Toxics and drugs were subsequently removed from the analysis.

The analysis of the study participants, as well as the changes in the nutrients and food groups intake after the intervention, together with the correlation analyses and linear regression models, was carried out with STATA 15.1 (Statacorp LP, College Station, TX, USA). The normality of the data was assessed using the Shapiro–Wilk test. The potential differences between groups at baseline were assessed using the appropriate statistical analyses (Student's *t*‐test, Mann–Whitney *U* test, or Chi‐square test). As multiple secondary outcomes appeared, a correction for multiple testing based on the Benjamini–Hochberg procedure was applied (Li et al., 2017). Correlations between the changes that occurred in the intervention and the putative metabolites and dietary variables (diet quality, food groups, and nutrient intake) were evaluated with Pearson or Spearman tests, depending on the normality of the variable. The identification of putative metabolites predicting the change in some other health‐related variables was performed through generalized estimating equation models, considering the intra‐cluster correlation between siblings.

## 3 RESULTS

### 3.1 Baseline characteristics of study participants

**FIGURE 1:**
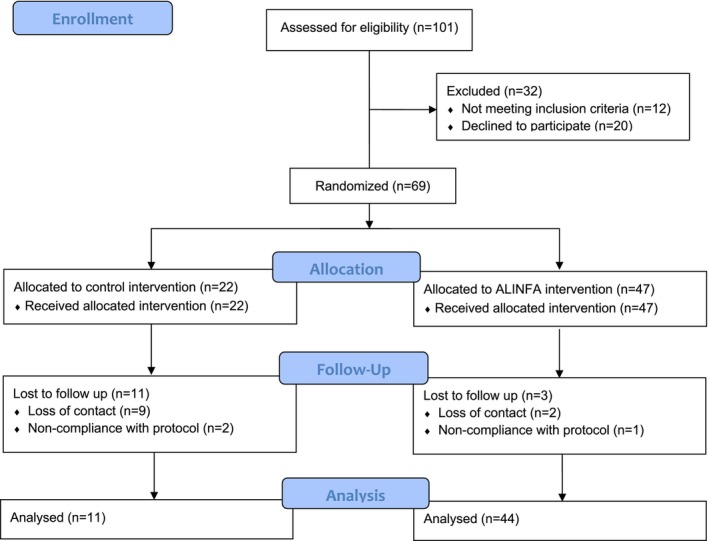
Allocation of subjects of the ALINFA Study according to the CONSORT 2010.

The participant flowchart is presented in Figure 1. A total of 69 children enrolled in the study and were randomly assigned in a 1:2 ratio to the control group (*n* = 22) or the ALINFA group (*n* = 47). Finally, 55 children completed the intervention, 11 in the control group, and 44 in the ALINFA group.

Baseline characteristics of the study population are shown in Table 1. No significant differences were observed between groups at baseline. The final sample consisted of 54.5% girls and 45.5% boys, and the average age of the study sample was 9.07 ± 1.73 years. The mean KIDMED index score at baseline was 6.90 ± 2.11 points in the control group and 7.06 ± 1.95 points in the ALINFA group, indicating in both cases the need for improvement in dietary pattern. The BMI *z*‐score in both the total population and in each group indicates appropriate weight/height for age.

**TABLE 1:** Baseline characteristics of children enrolled in the ALINFA study according to intervention group.

	All (*n* = 55)	Control (*n* = 11)	ALINFA (*n* = 44)	*p* Value^a^
%	100%	20%	80%	
Gender (boys/girls)	25/30	7/4	18/26	.176
Age (years)	9.07 ± 1.73	8.81 ± 1.53	9.12 ± 1.78	.590
Anthropometry				
Weight (kg)	34.84 ± 8.34	35.23 ± 8.76	34.74 ± 8.33	.863
Height (m)	1.38 ± 0.09	1.38 ± 0.09	1.38 ± 0.09	.928
BMI *z*‐score	0.09 ± 0.96	0.25 ± 0.93	0.05 ± 0.97	.554
Waist (cm)	61.62 ± 7.51	62.01 ± 7.66	61.52 ± 7.55	.924
SBP (mmHg)	101.9 ± 10.12	104.0 ± 10.75	101.4 ± 10.01	.155
DBP (mmHg)	67.24 ± 9.03	67.86 ± 9.63	67.0 ± 8.98	.802
Body composition				
Fat mass (kg)	7.93 ± 4.12	8.33 ± 4.42	7.82 ± 4.09	.808
Lean mass (kg)	26.86 ± 4.84	26.9 ± 4.79	26.85 ± 4.91	.975
Muscular mass (kg)	25.42 ± 4.60	25.46 ± 4.59	25.41 ± 4.66	.975
Diet quality (KIDMED index)				
Total punctuation	7.03 ± 1.97	6.90 ± 2.11	7.06 ± 1.95	.813
Interpretation				
Low diet quality	4 (7.27%)	1 (9.10%)	3 (6.82%)	.911^b^
Need to improve dietary pattern	28 (50.91%)	5 (45.45%)	23 (52.27%)	
Optimal MD	23 (41.82%)	5 (45.45%)	18 (40.91%)	
*Biochemistry*				
Total cholesterol (mg/dL)	173.5 ± 23.46	175.7 ± 25.28	172.9 ± 23.31	.752
HDL‐c (mg/dL)	62.26 ± 9.44	64.94 ± 13.25	61.51 ± 8.18	.341
LDL‐c (mg/dL)	99.88 ± 21.51	99.89 ± 21.46	99.87 ± 21.91	.998
Glucose (mg/dL)	93.21 ± 5.44	93.84 ± 3.62	93.03 ± 5.89	.698
Insulin (μIU/mL)	9.76 ± 4.03	8.88 ± 2.75	10.00 ± 4.33	.467
TNF‐α (pg/mL)	5.07 ± 0.98	4.94 ± 0.96	5.11 ± 1.00	.642
Leptin (ng/mL)	1.72 ± 1.37	1.88 ± 1.23	1.68 ± 1.42	.369
CRP (mg/dL)	0.87 ± 0.90	1.05 ± 1.24	0.82 ± 0.80	.752
IL‐6 (pg/mL)	24.26 ± 51.66	15.50 ± 41.46	26.72 ± 54.51	.075

*Note*: Data are mean ± SD.

^a^ *p* Values based on Student's *t*‐test or Mann–Whitney *U*.

^b^ *p* Values based on Chi‐square test. Statistical significance was defined as *p* < .05.

### 3.2 Analyses of nutrient intake and food group consumption in the ALINFA group

Tables 2 and 3 show changes in nutrient intake and food group consumption, respectively, after the intervention assessed by the FFQ. In the ALINFA group, a significant decrease in energy intake (*p* = .046), total lipids (*p* = .016) and saturated lipids (*p* = .011) was observed after the intervention. Also, an increase in fiber intake (*p* < .001) was reported at the end of the study.

**TABLE 2:** Difference in nutrient intake before and after the intervention in the ALINFA group (*n* = 44).

	Pre‐intervention	Post‐intervention	*p* Value
Kcal	2157 ± 378.7	1981 ± 367.0	.046
Total carbohydrates (g)	219.6 ± 41.75	209.7 ± 47.33	.389
Total lipids (g)	95.85 ± 19.69	87.06 ± 17.59	.016
Saturated	26.22 ± 7.38	22.51 ± 7.62	.011
Monounsaturated	39.77 ± 10.24	36.08 ± 6.12	.076
Polyunsaturated	11.66 ± 2.66	11.34 ± 2.57	.670
Total protein (g)	96.20 ± 31.73	89.63 ± 18.50	.737
Fiber (g)	20.84 ± 5.85	25.05 ± 6.30	.001
Cholesterol (mg)	288.41 ± 75.75	258.12 ± 81.81	.121
Sodium (mg)	3195 ± 793	3015 ± 814	.562

*Note*: Data are mean ± SD. Benjamini–Hochberg adjustment was applied. *p* Values based on Student's *t*‐test or Wilcoxon test. Statistical significance defined as *p* < .05.

**TABLE 3:** Difference in the consumption (g/day) of the main food groups before and after the intervention in the ALINFA group (*n* = 44).

	Pre‐intervention	Post‐intervention	*p* Value
Whole dairy (g/day)	356.1 ± 284.6	337.15 ± 235.9	.514
Low‐free fat dairy (g/day)	104.1 ± 131.1	105.07 ± 190.0	.732
Egg (g/day)	20.88 ± 8.81	20.26 ± 7.92	.806
Lean meat (g/day)	100.5 ± 36.02	110.4 ± 48.41	.308
Fatty meat (g/day)	37.61 ± 19.65	25.83 ± 27.34	.014
White fish (g/day)	14.72 ± 8.10	21.00 ± 8.76	.001
Fatty fish (g/day)	13.84 ± 10.66	14.25 ± 11.13	.772
Vegetable (g/day)	234.1 ± 109.6	226.3 ± 84.7	.847
Fruit (g/day)	328.5 ± 216.1	320.9 ± 142.2	.260
Pulse (g/day)	27.01 ± 11.29	33.19 ± 16.15	.004
Refined grains (g/day)	105.0 ± 43.06	81.8 ± 36.37	.008
Whole grains (g/day)	3.13 ± 8.08	46.72 ± 50.55	<.001
Nut (g/day)	6.08 ± 6.86	12.29 ± 7.95	<.001
Olive oil (g/day)	23.38 ± 9.53	21.79 ± 7.83	.741
Other fats (g/day)	14.93 ± 20.44	9.08 ± 11.26	.195
Pastries/ confectionery (g/day)	25.08 ± 12.75	13.03 ± 12.87	<.001
Fast food (g/day)	56.72 ± 20.44	39.44 ± 19.96	<.001
Sugars (g/day)	79.76 ± 64.44	51.29 ± 59.58	.001
Sweetened foods (g/day)	7.17 ± 19.92	3.93 ± 8.76	.104

*Note*: Data are mean ± SD. Benjamini–Hochberg adjustment was applied. *p* Values based on Student's *t*‐test or Wilcoxon test. Statistical significance defined as *p* < .05.

Regarding the consumption of food groups, after the intervention, the ALINFA group showed a significant increase in the intake of the following food groups: whole grains (*p* < .011), pulses (*p* = .004), nuts (*p* < .001), and white fish (*p* = .001). Likewise, a significant decrease in the consumption of refined grains (*p* = .008), fatty meat (*p* = .014), fast food (*p* < .001), pastries and confectionery (*p* < .001), and sugars (*p* = .001) was reported.

### 3.3 Urinary metabolomic profile

Pairwise analyses identified 6074 and 4760 features for the control group and the ALINFA group, respectively, in the ESI+ mode. In the ESI− mode, we identified 2829 features in the control group and 2000 features in the ALINFA group. In turn, of the total features identified in the ESI+ mode, 744 were found only in the ALINFA group, 2058 in the control group, and 4016 were common to both groups. Likewise, out of the total features identified in the ESI− mode, 465 were found only in the ALINFA group, 1294 in the control group, and 1535 were common to both groups. Thus, the discriminant features of the ALINFA group to be analyzed were identified.

**FIGURE 2:**
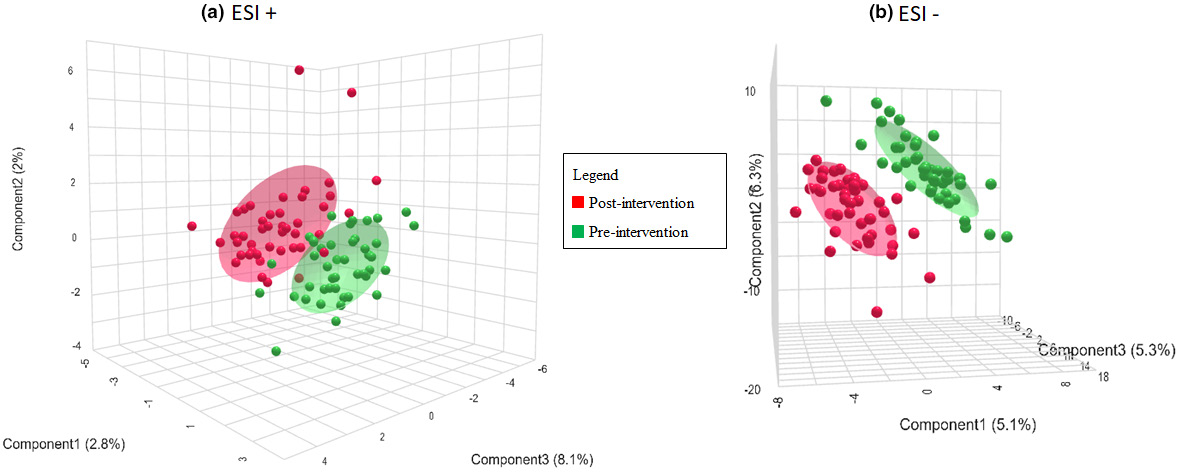
PLS‐DA score plots of the ALINFA group, including pre‐intervention and post‐intervention data. (a) PLS‐DA in the positive ionization mode (ESI+) (b) PLS‐DA in the negative ionization mode (ESI−).

**FIGURE 3:**
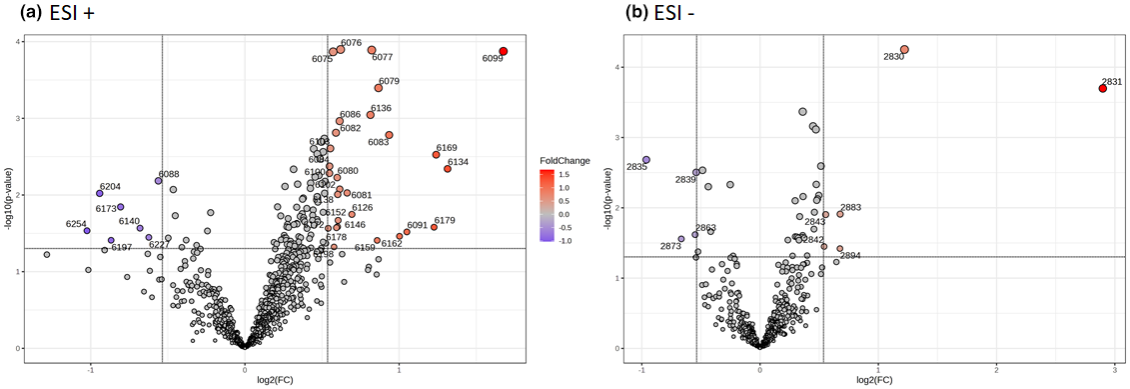
Volcano plot of the ALINFA group showing those discriminating metabolites that increased or decreased after the intervention. (a) Positive ionization mode (ESI+) (b) Negative ionization mode (ESI−).

The PLS‐DA approach was able to discriminate between two groups in both positive and negative ionization modes, as illustrated by the PLS‐DA score plots (Figure 2). For the PLS‐DA calculations, the accuracy, *R* ^2^ and *Q* ^2^, and permutation parameters were calculated. The values were accuracy = 0.856, *R* ^2^ = 0.618, and *Q* ^2^ = 0.470 based on 2 components and *p* < .001 in ESI+. Accuracy = 0.897, *R* ^2^ = 0.756 and *Q* ^2^ = 0.611 based on 2 components and *p* < .001 in ESI−. This indicates that the PLS‐DA model is robust and not overtrained. In the permutation tests, a total of 1000 resamplings were performed and calculated to determine the statistical significance. Therefore, urine samples from the ALINFA group were grouped according to study visit, pre‐intervention, and post‐intervention. This shows the compliance of the volunteers with the intervention, given the change in metabolite excretion after the study. Also, the PLS‐DA model was used to identify those features that discriminate between groups using the VIP score. In this case, a VIP score greater than 1 was determined. Finally, the Volcano plot was used to select the discriminant metabolites of the ALINFA intervention, selecting a *p*‐value < .05 and FC of 1.45 (Figure 3). Thus, 35 features were found in the ESI+ mode and 22 features in the ESI− mode. After removing those considered as toxic or drugs, 35 metabolites were identified in the ESI+ mode, of which 7 increased their concentration after the intervention and 28 decreased, and in the ESI− mode 10 metabolites were identified, of which 6 increased their concentration after the intervention and 4 decreased.

### 3.4 Identification of putative metabolites

Metabolites were approximately identified using information from multiple databases, in particular the Metabolomics Workbench database. Potential metabolites were identified as those in which the difference between the detected and theoretical mass values did not exceed 5 mDa. The information on all the potential metabolites identified is presented in Data S1, while Table 4 shows the information on the most relevant metabolites associated with this intervention, 11 of the ESI+ mode and 1 of the ESI− mode. Regarding the regulation of these 12 discriminating metabolites of the ALINFA intervention, 9 of them decreased after the intervention and 3 increased. Data on the mean intensity of each metabolite at baseline and after the end of the intervention, the VIP value, the retention time, the detected mass, the putative metabolite identification, the assignment, and the mass difference are presented in Table 4.

**TABLE 4:** Putative metabolites between the baseline and the end of the intervention in ESI+ and ESI− mode in the ALINFA group.

	Pre‐intervention	Post‐intervention	VIP value	RT (min)	Detected mass (m/z)	Putative metabolites	Assignation	Mass difference (mDa)
ESI+								
1	17.28	16.73	3.059	6.65	215.0602	Ser‐Ser	[M+Na]^+^	−3.60
2	15.00	13.89	2.719	9.48	283.2294	3‐oxo‐2‐pentylcyclopentane‐1‐hexanoic acid methyl ester	[M+H]^+^	2.63
3	17.54	16.76	1.894	8.42	288.1157	N‐Ribosylhistidine	[M+H]^+^	−3.24
4	14.05	13.22	1.944	7.15	717.3597	Amataine	[M+H]^+^	−4.94
5	18.35	17.85	2.247	8.25	210.0560	Indoleacrylic acid	[M+Na]^+^	3.49
6	13.95	13.30	1.813	7.79	318.1875	Suberoyl‐L‐carnitine	[M+H]^+^	−3.61
7	22.04	21.17	2.221	12.93	595.3436	7‐Dehydrodichapetalin E	[M+H]^+^	1.81
8	15.11	14.33	2.496	6.07	254.1475	Gly‐Pro‐Val	[M+H−H_2_O]^+^	−2.35
9	18.60	18.26	2.105	9.60	203.1100	6‐Acetyl‐2,2‐dimethyl‐2H‐1‐benzopyran	[M+H]^+^	3.25
10	18.70	19.43	1.630	6.28	143.0808	1,2,4‐Tris(methylene)cyclohexane	[M+Na]^+^	−2.28
11	16.67	17.69	1.941	10.08	380.10	S‐Lactoylglutathione	[M+H]^+^	−2.72
ESI−								
12	14.40	14.72	2.136	9.76	247.1232	Brasilidine A	[M−H]^−^	−0.89

*Note*: The data in pre‐intervention and post‐intervention column refer to the mean intensity of metabolites and are presented as log 2.

Abbreviation: RT, retention time.

The discriminating compounds selected in many cases presented various possible putative metabolites. In those cases, those metabolites considered as most probable were selected based on the existing bibliography and the characteristics of the intervention, thus being able to select the most appropriate markers of it. Of the 12 metabolites indicated as the most relevant, some of them were products derived from protein and aminoacid metabolism, such as N‐Ribosylhistidine, indoleacrylic acid, and different dipeptides and tripeptides, as well as metabolites from lipid metabolism (3‐oxo‐2‐pentylcyclopentane‐1‐hexanoic acid methyl, Suberoyl‐L‐carnitine, and 7‐Dehydrodichapetalin E) and pyruvate metabolism (S‐lactoylglutathione). In addition, other observed compounds were derivatives of benzene (6‐acetyl‐2,2‐dimethyl‐2H‐1‐benzopyran), alkaloids (Amataine and Brasilidine A), and branched unsaturated hydrocarbons (1,2,4‐Tris(methylene)cyclohexane).

### 3.5 Associations between putative metabolites and dietary variables

In order to further investigate possible associations between the urinary metabolites identified and the diet, a correlation study was carried out between the discriminating metabolites identified and the following dietary variables: nutrient intake, food group intake, and diet quality.

**FIGURE 4:**
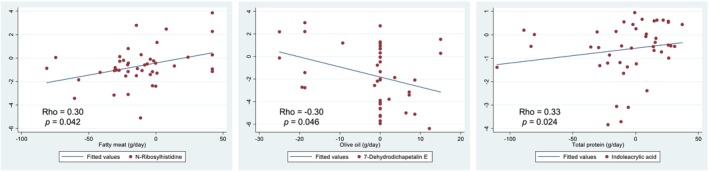
Correlation between putative metabolites and dietary intake variables (foods groups and nutrient intake) assessed by FFQ in the ALINFA group.

The correlation scatterplots between putative metabolites and nutrient and food group intake are shown in Figure 4. All correlated variables refer to the change after the intervention in the ALINFA group. The food groups that showed a significant correlation were fatty meat and olive oil. The change in fatty meat intake was positively associated with N‐Ribosylhistidine. On the other hand, olive oil intake was negatively associated with 7‐Dehydrodichapetalin E. Regarding nutrient intake, only a significant correlation was observed; a positive association was observed between total protein intake and indoleacrylic acid.

**FIGURE 5:**
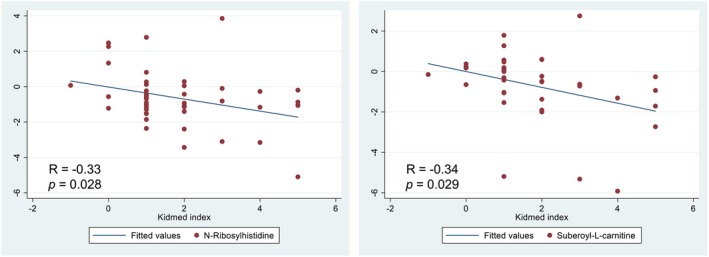
Correlation between putative metabolites and diet quality assessed by the KIDMED index in the ALINFA group.

Likewise, significant correlations were found between diet quality evaluated by the KIDMED index and two of the discriminating metabolites identified (Figure 5). Specifically, a negative association was observed between diet quality and N‐Ribosylhistidine and Suberoyl‐L‐carnitine. Finally, a linear regression model was built with these two metabolites to predict the change in diet quality (Table 5). This model was adjusted for caloric intake. In addition, the potential correlation between siblings was considered.

**TABLE 5:** Linear regression models of diet quality based on the KIDMED index questionnaire.

	KIDMED index		
	*β*	95% CI	*p* Value
N‐Ribosylhistidine	−.252	−0.457 to −0.047	.016
Suberoyl‐L‐carnitine	−.345	−0.619 to −0.071	.013
Kcal	−.0009	−0.001 to −0.0001	.014

*Note*: *β* represents changes in outcomes for an increasing number of units of the KIDMED index.

## 4 DISCUSSION

The present study investigates the effect of the ALINFA nutritional intervention on the urinary metabolomic profile in children 6 to 12 years old. The results show the change in discriminant metabolites after 2 months of following a full‐fixed meal plan. These changes in the urinary metabolome support the effectiveness of the ALINFA diet, as they demonstrate the effect of the dietary modifications carried out.

Twelve metabolites were identified as the most relevant biomarkers of the changes produced by this intervention. Many of them were associated with protein and amino acid metabolism. A decrease in the metabolites Ser‐Ser (dipeptide), N‐Ribosylhistidine (histidine derivatives), indoleacrylic acid (a metabolite produced by microorganisms from tryptophan), and Gly‐Pro‐Val (tripeptide) was observed in the ALINFA group after the intervention. These metabolites were mainly associated with animal protein, especially fatty meat. This finding reflects changes in the type and amount of protein‐rich foods consumed, as observed in changes in nutrient intake after the intervention. In addition, bacterial products of protein degradation, such as Indoleacrylic acid, are generally associated with detrimental effects (Roager & Licht, 2018). Likewise, a systematic review showed that a higher concentration of tryptophan‐derived metabolites was linked to obesity (Handakas et al., 2022), and in a trial carried out in adult women, a greater amount of indole metabolites was found in those women with obesity (Ding et al., 2021). Despite these results, recent research suggests that microbial tryptophan catabolites may also have a beneficial effect on the physiology of the host (Agus et al., 2018; Roager & Licht, 2018).

Metabolites of lipid fatty acid metabolism were also detected, indicating potential positive changes in the diet. The three metabolites identified, 3‐oxo‐2‐pentylcyclopentane‐1‐hexanoic acid methyl (eicosanoid), Suberoyl‐L‐carnitine (acylcarnitine), and 7‐Dehydrodichapetalin E (sterol), decreased after the intervention. 3‐oxo‐2‐pentylcyclopentane‐1‐hexanoic acid methyl is a metabolite of the eicosanoid group, which are oxidized derivatives of 20‐carbon polyunsaturated fatty acids, mainly arachidonic acid (Davies, 2008). It is a metabolite that appears to be related to the prostaglandin group of eicosanoids. Arachidonic acid is obtained from foods such as poultry, animal meat, fish, seafood, and eggs (Calder, 2020). Besides, 7‐Dehydrodichapetalin E is a cholesterol and derivate metabolite. Cholesterol is found in food sources of animal origin, the main ones being eggs, meats (particularly red and processed meats), seafood, and dairy products (Cha & Park, 2019). Given the observed decrease in red meat consumption, this could explain the decrease of these two metabolites in urine. In addition, the intervention also showed a decrease in total fat intake, which could also have resulted in this reduction. Another metabolite that showed a decrease was suberyl‐L‐carnitine, which is a medium‐chain acylcarnitine responsible for transporting fatty acids into the mitochondria. These compounds are formed from carnitine, which can be biosynthesized by the body itself or obtained from the diet. One of the main food sources of this compound is red meat (Pekala et al., 2011). Consumption of red and processed meats is associated with increased urinary excretion of acylcarnitines, according to recent studies (Khodorova et al., 2019; Wedekind et al., 2020), as observed in this study, in which the reduction in the consumption of this type of meat was translated into a reduction in this type of compound. Moreover, this finding agrees with the results observed for the metabolites of protein metabolism. In addition, it has been observed that short‐chain acylcarnitines are elevated in the urine and serum of patients with obesity (Dambrova et al., 2022; Mihalik et al., 2010; Wang et al., 2011) and in the blood serum of those with diabetes (Mihalik et al., 2010).

In addition, in relation to pyruvate metabolism, it was found that S‐Lactoylglutathione increased after the intervention. S‐Lactoylglutathione is a metabolite that is formed through the reaction between lactate and glutathione. Glutathione (gamma‐glutamyl‐cysteinyl‐glycine) is an antioxidative molecule that plays a key role in protecting against oxidative stress (Marí et al., 2020). This metabolite can be found in a wide variety of food sources, including fruits, vegetables, cereals, legumes, and nuts. Given the results observed in the consumption of food groups, it is possible that this increase is due to the higher consumption of legumes and nuts.

Another compound found was a benzene derivative, 6‐Acetyl‐2,2‐dimethyl‐2H‐1‐benzopyran (benzopyran), that decreased after the intervention. This compound has been detected and is related to fats and oils. Specifically, the food source in which it was found in the highest concentrations was sunflower. These data are once again consistent with those observed in the consumption of food groups, since a decrease in the consumption of fats other than olive oil, such as sunflower, among others, was observed.

Alkaloids and metabolites were also found in the ALINFA group, showing contradictory results. Amataine decreased after the intervention, while Brasilidine A increased. Both metabolites belong to the group of indole alkaloids. Plant‐based indol alkaloids are known for their multiple pharmacological activities, such as anticancer, anti‐inflammatory, antidepressant, analgesic, hypotensive, hypolipidemic, antidiabetic, and many others (Omar et al., 2021). Indole derivatives are often found in cruciferous vegetables such as broccoli, cabbage, and Brussels sprouts (Dhuguru & Skouta, 2020). Furthermore, 1,2,4‐Tris(methylene)cyclohexane, a branched unsaturated hydrocarbon, increased after the intervention. This metabolite has been reported as a biomarker of fruit consumption. Despite the fact that that food group consumption data did not show an increase in fruit intake, the metabolomics results seem to indicate that there was an increase in fruit consumption.

Correlation analyses showed associations between the consumption of nutrients and food groups and the metabolites found in urine. These results made it possible to confirm the previous associations identified in the literature. Regarding protein intake, two positive associations were confirmed. The association between the intake of fatty meat and the putative metabolite 3, which is likely to be the compound N‐Ribosylhistidine, was demonstrated. Also, the direct association between the amount of protein intake and the putative metabolite 5 was demonstrated, which is probably Indoleacrylic acid. Regarding lipid intake, a negative association was observed between putative metabolite 7 and olive oil intake. This metabolite is likely to be 7‐Dehydrodichapetalin E, although this compound has not been associated with this food in the literature, since it is found in foods of animal origin. This association, although not direct, may be due to the change in the type of fat consumed, a change to a lower consumption of saturated fat, and an increase in the consumption of healthy fats.

Furthermore, a negative correlation was observed between two metabolites and diet quality, putative metabolite 3 and 6 (N‐Ribosylhistidine and Suberoyl‐L‐carnitine respectively). Both putative metabolites are related to the intake of fatty and red meat and, therefore, saturated fat. In addition, the regressions carried out allowed us to identify these two metabolites as the most important biomarkers of this intervention, since the main objective of the ALINFA study was to improve the quality of the diet.

This study has some limitations. First, the sample size is relatively small. Second is the lack of bibliographic references on many of the metabolites found and on studies carried out in children that analyze the urinary metabolomic profile. Finally, the findings of the untargeted metabolomic analysis should be further validated with targeted approaches. In contrast, this study has several strengths. First, it is one of the few randomized and controlled trials carried out in Spanish children that evaluate the urinary metabolomic changes produced after a nutritional intervention. Second, a validated questionnaire for the pediatric population was obtained to assess dietary intake and food groups. Lastly, the fact that this study was to ensure the food supply increases the reliability of the observed results.

## 5 CONCLUSION

In conclusion, the changes observed in the urinary metabolomic profile demonstrate the efficacy of the ALINFA nutritional intervention, derived from the positive changes observed at the dietary level. Following a nutritional plan for 2 months is time enough to observe changes in urinary metabolites. In addition, the results show possible useful biomarkers of dietary intake and diet quality. Further research is needed to fully understand the implications of these metabolomic changes as well as to develop effective dietary interventions in children in the long term to improve their dietary pattern.

## AUTHOR CONTRIBUTIONS

**Naroa Andueza:** Conceptualization (equal); data curation (equal); formal analysis (equal); investigation (equal); writing – original draft (equal). **David Muñoz‐Prieto:** Formal analysis (equal); methodology (equal); software (equal); validation (equal); writing – review and editing (equal). **Ana Romo‐Hualde:** Investigation (equal); methodology (equal); visualization (equal); writing – review and editing (equal). **Marta Cuervo:** Conceptualization (equal); formal analysis (equal); funding acquisition (supporting); investigation (equal); project administration (equal); validation (equal); visualization (equal); writing – review and editing (equal). **Santiago Navas‐Carretero:** Conceptualization (equal); data curation (equal); funding acquisition (lead); investigation (equal); methodology (equal); resources (equal); validation (equal); writing – review and editing (equal).

## FUNDING INFORMATION

This research was funded by the Government of Navarra (ALINFA, ref. 0011‐1411‐2019‐000033). A research contract (N.A.) was granted by the Center for Nutrition Research of the University of Navarra.

## CONFLICT OF INTEREST STATEMENT

The authors declare that they have no conflict of interest.

## ETHICAL STATEMENT

The study was approved by the Research Ethics Committee of the University of Navarra (ref. 2021.027) and was conducted in accordance with the Declaration of Helsinki.

## INFORMED CONSENT

Written informed consent was obtained from all study participants.

## Supporting information


Data S1.

## Data Availability

Data are available on request from the corresponding author.
